# Supplementary material for: Temporal requirements of SKN-1/NRF as a regulator of lifespan and proteostasis in Caenorhabditis elegans
Source: PLoS One. 2021 Jul 1;16(7):e0243522. doi: 10.1371/journal.pone.0243522 (PMC8248617; doi:10.1371/journal.pone.0243522)
Supplement: S2 Fig — (PDF) [file pone.0243522.s002.pdf]

Supplemental figure 2

A

Age-associated paralysis in wild type worms

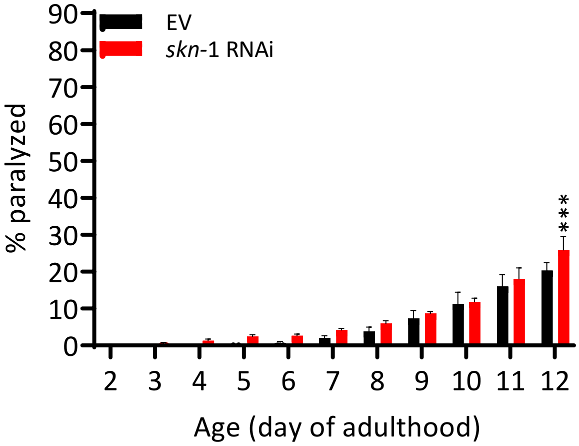

***P≤0.001

Strain: N2
Error Bars ± SEM
N=5

B

skn-1 reduction enhances paralysis in neurons

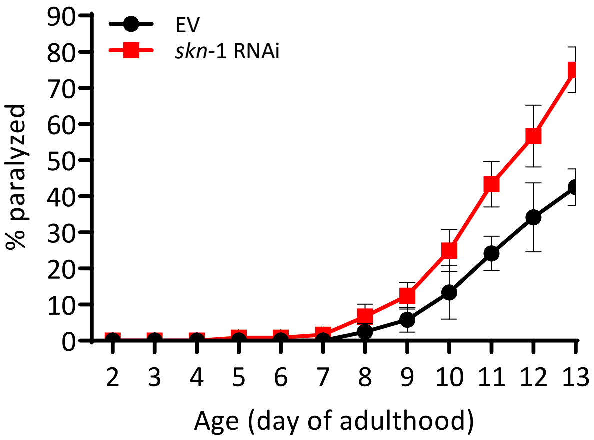

Strain: AGD1246
Error Bars ± SEM

C

skn-1 reduction enhances paralysis in neurons

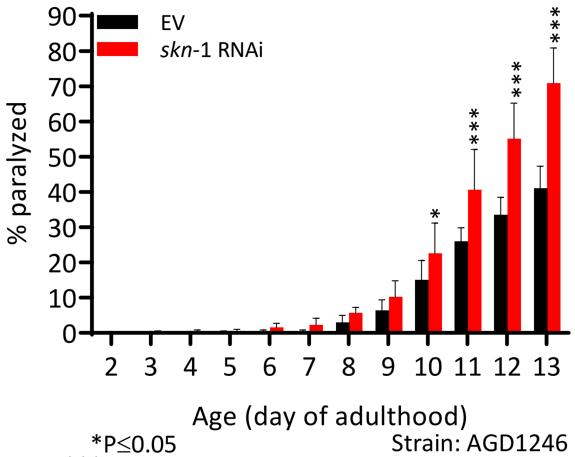

*P≤0.05
***P≤0.001

Strain: AGD1246
Error Bars ± SEM
N=4
