# Supplementary figures and images for: Temporal requirements of SKN-1/NRF as a regulator of lifespan and proteostasis in Caenorhabditis elegans
Source: PLoS One. 2021 Jul 1;16(7):e0243522. doi: 10.1371/journal.pone.0243522 (PMC8248617; doi:10.1371/journal.pone.0243522)

Supplemental figure 3

An illustration of temporal application of RNAi

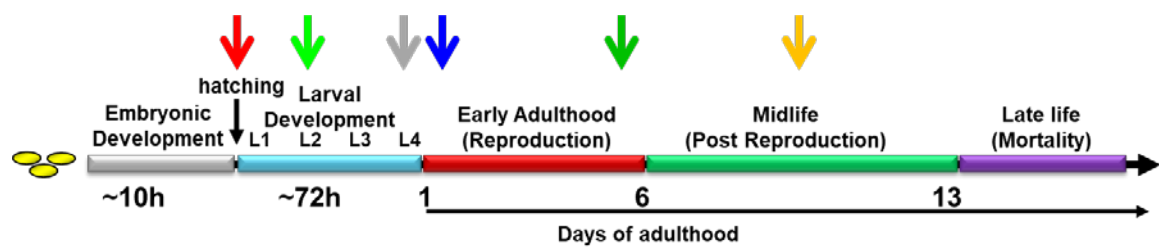

Supplement: S3 Fig — (PDF) [file pone.0243522.s003.pdf]

Supplemental figure 4

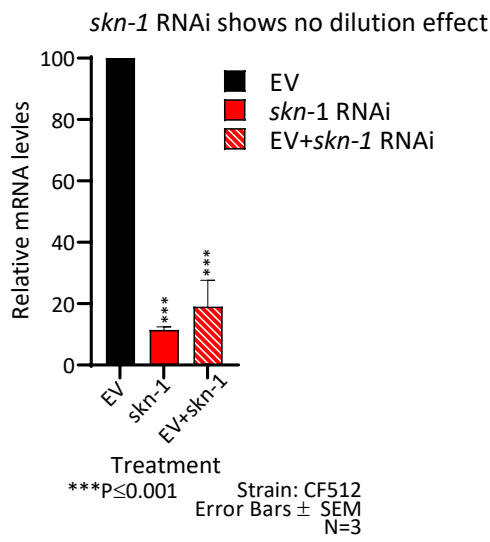

Supplement: S4 Fig — (PDF) [file pone.0243522.s004.pdf]

Supplemental figure 6

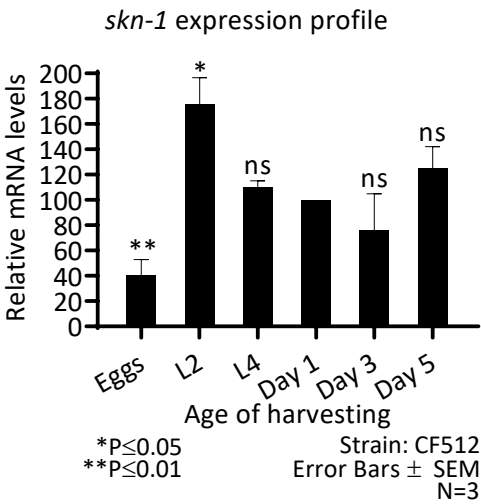

Supplement: S6 Fig — (PDF) [file pone.0243522.s006.pdf]
