# Supplementary material for: Temporal requirements of SKN-1/NRF as a regulator of lifespan and proteostasis in Caenorhabditis elegans
Source: PLoS One. 2021 Jul 1;16(7):e0243522. doi: 10.1371/journal.pone.0243522 (PMC8248617; doi:10.1371/journal.pone.0243522)
Supplement: S1 Table — (PDF) [file pone.0243522.s007.pdf]

Supplemental Table 1
Lifespan of N_2 worms that were treated with *skn-1* RNAi from different stages of life. (Corresponding to Fig. 1A and B)
Strain: N2

Treatment:	*n*	Censored:	Mean lifespan (days)	Standard error (days)	P value compared to control (EV)
EV	76	44	18.12	0.51	
skn-1 RNAi	106	14	16.17	0.32	4.09E-04
EV → *skn-1* RNAi (L2)	99	21	15.98	0.24	3.00E-05
EV → *skn-1* RNAi (L4)	74	46	15.54	0.35	2.60E-05
EV → *skn-1* RNAi (Day 1)	108	12	17.83	0.31	0.31
EV → *skn-1* RNAi (Day 5)	70	50	17.51	0.42	0.18
EV → *skn-1* RNAi (Day 9)	82	38	18.57	0.54	0.27
