# Supplementary material for: Temporal requirements of SKN-1/NRF as a regulator of lifespan and proteostasis in Caenorhabditis elegans
Source: PLoS One. 2021 Jul 1;16(7):e0243522. doi: 10.1371/journal.pone.0243522 (PMC8248617; doi:10.1371/journal.pone.0243522)
Supplement: S2 Table — A: Numerical data of a lifespan experiment presented at Fig 1C. B: Numerical data of a lifespan experiment of CF512 worms treated throughout life with EV or skn-1 RNAi or transferred from EV bacteria onto skn-1 RNAi at day 1 of adulthood. (PDF) [file pone.0243522.s008.pdf]

**Supplemental Table 2A**
**Lifespan of CF512 worms that were treated with *skn-1* RNAi from day 1 of adulthood. (Corresponding to Fig. 1C)**

Strain: CF512

| Treatment: | *n* | Censored: | Mean lifespan (days) | Standard error (days) | P value compared to control (EV) |
|---|---|---|---|---|---|
| EV | 92 | 28 | 21.82 | 0.49 | |
| *skn-1* RNAi | 94 | 26 | 17.79 | 0.37 | 1.61E-10 |
| EV → *skn-1* RNAi (Day 1) | 83 | 37 | 19.94 | 0.42 | 2.01E-3 |

**Supplemental Table 2B**
**Lifespan of CF512 worms that were treated with *skn-1* RNAi from day 1 of adulthood. (Biological repeat)**

Strain: CF512

| Treatment: | *n* | Censored: | Mean lifespan (days) | Standard error (days) | P value compared to control (EV) |
|---|---|---|---|---|---|
| EV | 90 | 30 | 22.12 | 0.39 | |
| *skn-1* RNAi | 106 | 14 | 17.66 | 0.38 | 1.8E-14 |
| EV → *skn-1* RNAi (Day 1) | 82 | 38 | 20.68 | 0.29 | 2.05E-03 |
