# Supplementary material for: Temporal requirements of SKN-1/NRF as a regulator of lifespan and proteostasis in Caenorhabditis elegans
Source: PLoS One. 2021 Jul 1;16(7):e0243522. doi: 10.1371/journal.pone.0243522 (PMC8248617; doi:10.1371/journal.pone.0243522)
Supplement: S3 Table — A: Numerical data of a lifespan experiment presented at Fig 1D. B: Numerical data of a lifespan experiment of CB1370 worms treated throughout life with EV or skn-1 RNAi or transferred from EV bacteria onto skn-1 RNAi at day 1 of adulthood. (PDF) [file pone.0243522.s009.pdf]

Supplemental Table 3A

Lifespan of *e1370* worms that were treated with *skn-1* RNAi from day 1 of adulthood.
(Corresponding to Fig. 1D)

Strain: CB1370

Treatment:	*n*	Censored:	Mean lifespan (days)	Standard error (days)	P value compared to control (EV)
EV	56	64	44.79	1.97	
skn-1 RNAi	109	11	30.63	0.94	4.64E-12
EV → *skn-1* RNAi (Day 1)	104	16	37.02	1.02	7.88E-05

Supplemental Table 3B
Lifespan of *e1370* worms that were treated with *skn-1* RNAi from day 1 of adulthood. (Biological repeat)
Strain: CB1370

Treatment:	*n*	Censored:	Mean lifespan (days)	Standard error (days)	P value compared to control (EV)
EV	59	61	45.24	2.16	
skn-1 RNAi	98	22	30.18	1.02	2.5E-11
EV → *skn-1* RNAi (Day 1)	107	12	33.89	1.15	4.8E-07
