# Supplementary material for: Temporal requirements of SKN-1/NRF as a regulator of lifespan and proteostasis in Caenorhabditis elegans
Source: PLoS One. 2021 Jul 1;16(7):e0243522. doi: 10.1371/journal.pone.0243522 (PMC8248617; doi:10.1371/journal.pone.0243522)
Supplement: S4 Table — A: Numerical data of a lifespan experiment presented at Fig 1E. B: Numerical data of a lifespan experiment of DA1116 worms treated throughout life with EV or skn-1 RNAi or transferred from EV bacteria onto skn-1 RNAi at day 1 of adulthood. (PDF) [file pone.0243522.s010.pdf]

**Supplemental Table 4A**
**Lifespan of DA1116 worms that were treated with *skn-1* RNAi from day 1 of adulthood. (Corresponding to Fig. 1E)**

Strain: DA1116

| Treatment: | *n* | Censored: | Mean lifespan (days) | Standard error (days) | P value compared to control (EV) |
|---|---|---|---|---|---|
| EV | 68 | 52 | 20.70 | 0.90 | |
| *skn-1* RNAi | 97 | 23 | 14.93 | 0.35 | 1.32E-10 |
| EV → *skn-1* RNAi (Day 1) | 73 | 47 | 17.89 | 0.60 | 4.83E-3 |

**Supplemental Table 4B**
**Lifespan of DA1116 worms that were treated with *skn-1* RNAi from day 1 of adulthood. (Biological repeat)**

Strain: DA1116

| Treatment: | *n* | Censored: | Mean lifespan (days) | Standard error (days) | P value compared to control (EV) |
|---|---|---|---|---|---|
| EV | 74 | 46 | 19.09 | 0.77 | |
| *skn-1* RNAi | 91 | 29 | 15.27 | 0.36 | 3.75E-05 |
| EV → *skn-1* RNAi (Day 1) | 71 | 49 | 17.30 | 0.44 | 0.02 |
